# CD95/Fas suppresses NF-κB activation through recruitment of KPC2 in a CD95L/FasL-independent mechanism

**DOI:** 10.1016/j.isci.2021.103538

**Published:** 2021-12-01

**Authors:** Jean-Philippe Guégan, Justine Pollet, Christophe Ginestier, Emmanuelle Charafe-Jauffret, Marcus E. Peter, Patrick Legembre

**Affiliations:** 1 Explicyte, Cours de l’Argonne, 33000 Bordeaux, France; 2 Technological core facility BISCEm, Université de Limoges, US042 Inserm, UMS 2015 CNRS, Centre hospitalo-universitaire de Limoges, Limoges, France; 3 Aix Marseille University, CNRS, INSERM, Institut Paoli-Calmettes, CRCM, Molecular Oncology "Equipe labellisée Ligue Contre le Cancer", Marseille, France; 4 Division Hematology/Oncology, Department of Medicine, Feinberg School of Medicine, Northwestern University, Chicago, IL 60611, USA; 5 Contrôle de la Réponse Immune B et lymphoproliférations, CRIBL, Université Limoges, UMR CNRS 7276, INSERM 1262, Limoges, France

**Keywords:** Immunology, Cell biology, Cancer

## Abstract

CD95 expression is preserved in triple-negative breast cancers (TNBCs), and CD95 loss in these cells triggers the induction of a pro-inflammatory program, promoting the recruitment of cytotoxic NK cells impairing tumor growth. Herein, we identify a novel interaction partner of CD95, Kip1 ubiquitination-promoting complex protein 2 (KPC2), using an unbiased proteomic approach. Independently of CD95L, CD95/KPC2 interaction contributes to the partial degradation of p105 (NF-κB1) and the subsequent generation of p50 homodimers, which transcriptionally represses NF-κB-driven gene expression. Mechanistically, KPC2 interacts with the C-terminal region of CD95 and serves as an adaptor to recruit RelA (p65) and KPC1, which acts as E3 ubiquitin-protein ligase promoting the degradation of p105 into p50. Loss of CD95 in TNBC cells releases KPC2, limiting the formation of the NF-κB inhibitory homodimer complex (p50/p50), promoting NF-κB activation and the production of pro-inflammatory cytokines, which might contribute to remodeling the immune landscape in TNBC cells.

## Highlights

• CD95 inhibits the NF-κB pathway via the partial degradation of p105 (NF-κB1)
• CD95 loss in TNBC cells induces the secretion of inflammatory cytokines
• CD95 C-term region interacts with KIP1 ubiquitination-promoting complex (KPC)-2
• CD95 directly binds KPC2 (UBAC1), which serves as an adaptor for p65 and KPC1

## Graphical abstract

**Figure:**
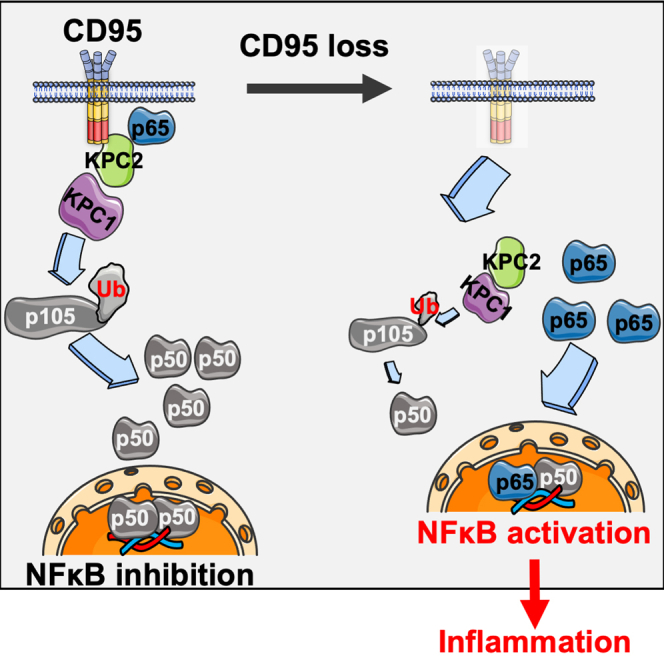

## Introduction

Among women, breast cancer (BC) is the most common cause of cancer and the second leading cause of cancer death (DeSantis et al., 2016). The molecular classification of BC distinguishes luminal A and B expressing estrogen (ER) and/or progesterone receptors (PR), basal/triple negative breast cancer (TNBC), and human epidermal growth factor receptor 2 (HER2)-like tumors. This molecular taxonomy is clinically relevant, with basal/TNBC patients presenting the poorest clinical outcome with no targeted therapies available when compared with other molecular subtypes. Interestingly, although CD95L (FasL/CD178)-expressing immune cells edit tumor cells by sparing cells expressing low CD95 (Fas/APO-1) level at their plasma membrane (Strasser et al., 2009), the complete loss of CD95 is deleterious to tumor growth (Chen et al., 2010). Interestingly, despite being resistant to CD95 mediated apoptosis, TNBCs maintain a very high amount of surface CD95 when compared with other breast cancers (Blok et al., 2017), and we recently found that CD95 loss in TNBC cells reprograms the immune landscape, by triggering a pro-inflammatory response unleashing the anti-tumor activity of natural killer (NK) cells (Qadir et al., 2021). The CD95-dependent molecular mechanism responsible for the recruitment and activation of NK cells in TNBC remains to be elucidated.

CD95 belongs to the tumor necrosis factor (TNF) receptor family and has mainly been viewed as a death receptor (Peter et al., 2015). However, recent data highlighted that this receptor can also induce nonapoptotic signaling pathways involved in physiological processes (Desbarats et al., 2003; Desbarats and Newell, 2000) or in the progression of auto-immune (O' Reilly et al., 2009; Poissonnier et al., 2016; Tauzin et al., 2011) and cancer disorders (Barnhart et al., 2004; Kleber et al., 2008). The ligand of CD95, CD95L, is a member of TNF superfamily that has been initially found expressed by activated T lymphocytes and NK cells to kill infected and transformed cells through cell-to-cell contact (Suda et al., 1993). CD95L extracellular domain can also be cleaved by metalloproteases (Fouque et al., 2014) to release a soluble CD95L (s-CD95L). Unlike membrane-bound CD95L (m-CD95L), s-CD95L fails to trigger cell death but instead contributes to aggravating inflammation in chronic inflammatory disorders such as systemic lupus erythematosus (SLE) (O' Reilly et al., 2009; Tauzin et al., 2011) and cancers (Barnhart et al., 2004; Hoogwater et al., 2010; Kleber et al., 2008; Malleter et al., 2013). The intracellular death domain (DD) of CD95 serves as a docking platform to trigger cell death. Upon binding of m-CD95L, CD95 recruits the adaptor protein Fas-associated death domain (FADD), which in turn aggregates the initiator caspase-8 and -10. The CD95/FADD/caspase complex is a designated death-inducing signaling complex (DISC) and contributes to the induction of the apoptotic program (Kischkel et al., 1995). Although s-CD95L fails to induce DISC, this ligand triggers the formation of a nonapoptotic complex termed motility-inducing signaling complex (MISC), which promotes migration of cancer cells (Kleber et al., 2008; Malleter et al., 2013; Tauzin et al., 2011). This nonapoptotic signal relies on the juxtamembrane region of CD95 that we designated calcium-inducing domain (CID). Finally, although the last 15 aa of CD95 have been reported to interact with the protein tyrosine phosphatase FAP-1 (Sato et al., 1995) or Dlg1 (Gagnoux-Palacios et al., 2018) to impair cell death, the biological role of this region remains poorly defined.

Using a BioID proteomic analysis, we have now identified Kip1 ubiquitylation-promoting complex 2 (KPC2) as a novel binding partner for CD95. KPC2 in turn recruits the ubiquitin ligase KPC1 and the Rel family member p65. Bound to CD95, the KPC2/KPC1 complex causes the ubiquitination of p105 and its partial degradation into p50. Loss of CD95 in TNBC favors the formation of the NF-κB transcriptomic activator p50/p65 heterodimer at the expense of its inhibitor counterpart p50/p50. In these otherwise CD95 signaling deficient cells, this promotes the induction of a pro-inflammatory NF-κB signaling pathway, resulting in the release of a set of cytokines known to recruit and activate multiple immune effector cells including NK cells.

## Results

### CD95 elimination in TNBC cells activates an inflammatory program

**Figure 1:**
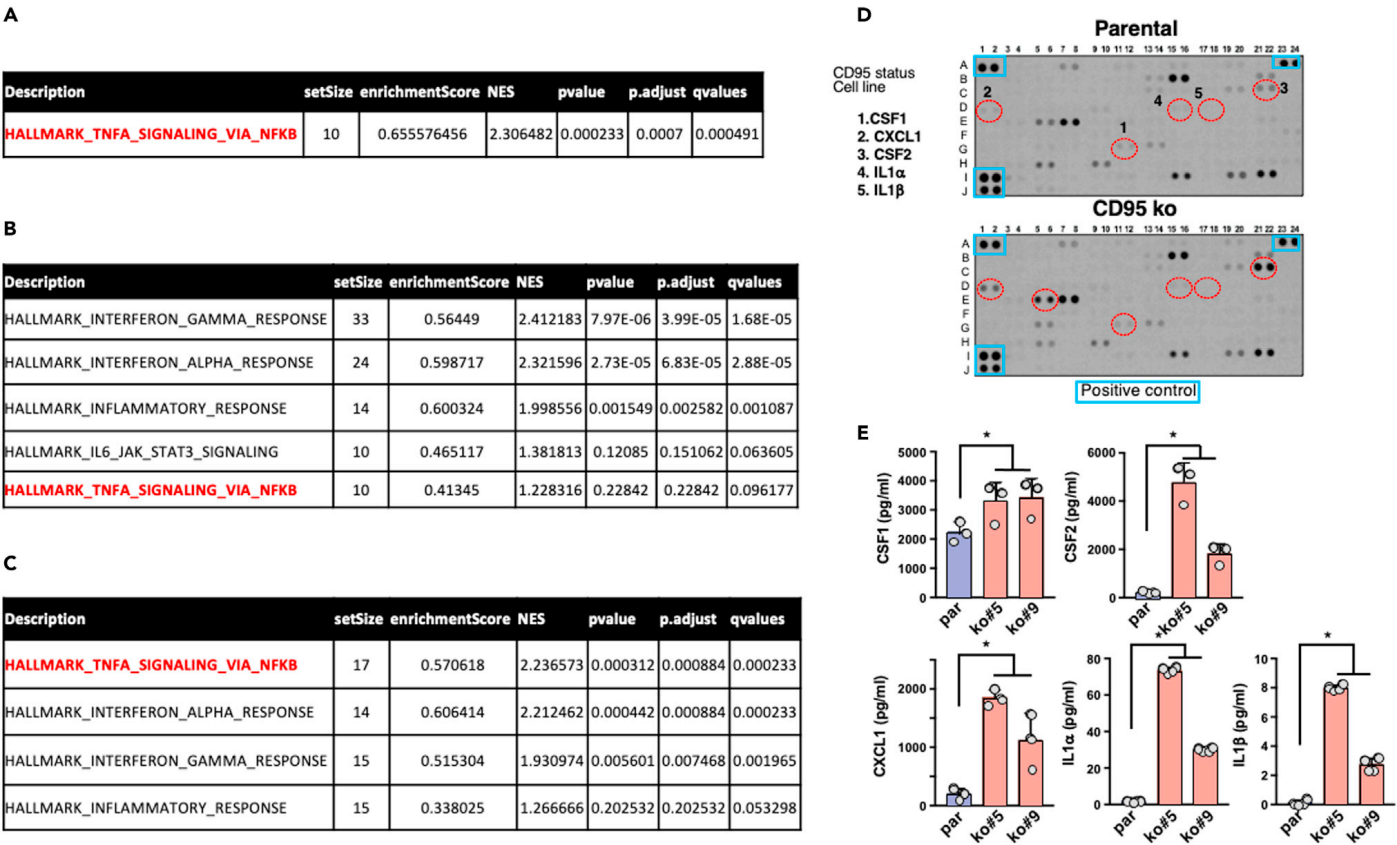
Loss of CD95 induces an inflammatory transcriptomic signature in TNBC cells (A) GSEA analysis performed on 386 genes (with FC ≥ 1.5 or FC ≤ −1.5 and p value ≤ 0.05) deregulated between parental TNBC MDA-MB-231 cell line and two CD95 k.o. counterparts. (B) GSEA analysis performed on 244 genes (with FC ≥ 1.5 or FC ≤ −1.5 and p.value ≤ 0.05) between two parental TNBC 4T1 clones and two CD95 k.o. counterparts. (C) GSEA analysis performed on 148 genes modulated in a similar fashion in mouse (4T1 cells) and human (MDA-MB-231 cells) TNBC cells and their respective CD95 KO cells. (D) The expression level of 105 different cytokines was evaluated using Proteome Profiler Human XL Cytokine Array Kit in supernatants of parental TNBC cells and their CD95 k.o. counterpart. Image is representative of three independently performed experiments. (E) Certain inflammatory cytokines upregulated in the Proteome Profiler Human XL Cytokine Array Kit in D and Table S4 were dosed by ELISA in supernatants of CD95 k.o. TNBC cells as compared with those of parental counterpart. Mean ± SD, p values were calculated using Nonparametric Mann-Whitney test (n = 3 or 4 for IL-1α and β cytokines).

Our data showing that loss of CD95 in TNBC cells promotes the remodeling of the immune landscape and triggers the anti-tumor activity of NK cells in a TNBC mouse model raised the question of whether CD95 loss by itself in TNBC cells could actively reprogram the transcriptomic signature of these cells. To address this question, we deleted CD95 in human (MDA-MB-231) (Figure S1A) and mouse (4T1) (Qadir et al., 2021) TNBC cells. Interestingly, the loss of CD95 in human (Table S1) and mouse (Table S2) TNBC cells induced the deregulation of 386 and 244 genes, respectively, with a fold-change cutoff of 1.5 (adjusted p < 0.05) between parental and CD95 k.o. TNBC cells. An analysis of the genes deregulated between wild-type and CD95 k.o. TNBC cells and shared between mouse (4T1 cell line) and human (MDA-MB-231 cell line (fold-change of 1.5 and adjusted p < 0.1) identified 148 genes (Table S3). To evaluate whether a biological process hallmark could be associated with the loss of CD95 in these analyses, we performed a Gene Set Enrichment Analysis (GSEA) using Molecular Signatures Database (MSigDB) (Liberzon et al., 2015). Interestingly, only the “TNFα signaling via NF-κB” hallmark was shared between the two TNBC cell lines in the three different analyses (see Tables S1, S2, and S3; Figures 1A–1C).

To validate the inflammatory signature associated with CD95 loss in TNBC cells, we conducted a comprehensive analysis of cytokines whose expression changed in CD95 k.o. MDA-MB-231 cells as compared with their parental counterpart (Figure 1D). Among the 105 secreted factors, a proteome profiler Human XL cytokine array revealed that 28 cytokines were overexpressed with a cut-off greater than or equal to 2 and 12 cytokines downregulated more than or equal to 2-fold (Figure 1D and Table S4). Quantitative (q)PCR (Figure S1B) and ELISAs confirmed the increased expression and secretion of cytokines known to promote the recruitment or the cytotoxic activity of NK cells including CSF1 (Thompson et al., 2018), CSF2 (Gillessen et al., 2003), IL1α (Lin et al., 2016), and IL1β (Cooper et al., 2001) or activate the inflammasome such as CXCL1 (Boro and Balaji, 2017) by CD95 k.o. MDA-MB-231 cells when compared with their wild-type counterparts (Figure 1E). Of note, no trace of soluble CD95L was detected in this assay (Table S4).

Although we failed to detect m-CD95L at the plasma membrane of human MDA-MB-231 and mouse 4T1 TNBC cells (Figure S1C), we could not rule out that an undetectable amount of m-CD95L still engaged CD95 in an autocrine fashion to induce basal signaling, which was abrogated by the loss of CD95 in cancer cells. To evaluate this possibility, we knocked-out CD95L in 4T1 TNBC cells. The selected clones harbored an inserted base (adenine) within the second CD95L codon, leading to a frameshift (Figure S1D). Unlike CD95-k.o. 4T1 cells (Figure S1E), CD95L-k.o. 4T1 cells did not exhibit a significant difference in CSF1, CSF2, CXCL1, IL1α, and IL1β transcription when compared with parental (Figure S1F), indicating that elimination of the ligand did not recapitulate the inflammatory signature observed with CD95 loss. To confirm this finding, we incubated for 24 h mouse 4T1 cells and human MDA-MB-231 cells with neutralizing anti-CD95L mAbs and then, analyzed the 5-genes pro-inflammatory signature. We used NOK-1 and MFL4 antibodies to selectively neutralize human and mouse CD95L, respectively. The transcription of the 5 pro-inflammatory genes remained unaffected in TNBC cells incubated with antibodies abrogating the CD95/CD95L interaction (Figure S1G). These findings suggested that the loss of CD95 in TNBC cells triggers a pro-inflammatory signaling pathway through a ligand-independent mechanism.

### A NF-**κ**B-dependent signaling pathway is activated in CD95 k.o. TNBC cells

**Figure 2:**
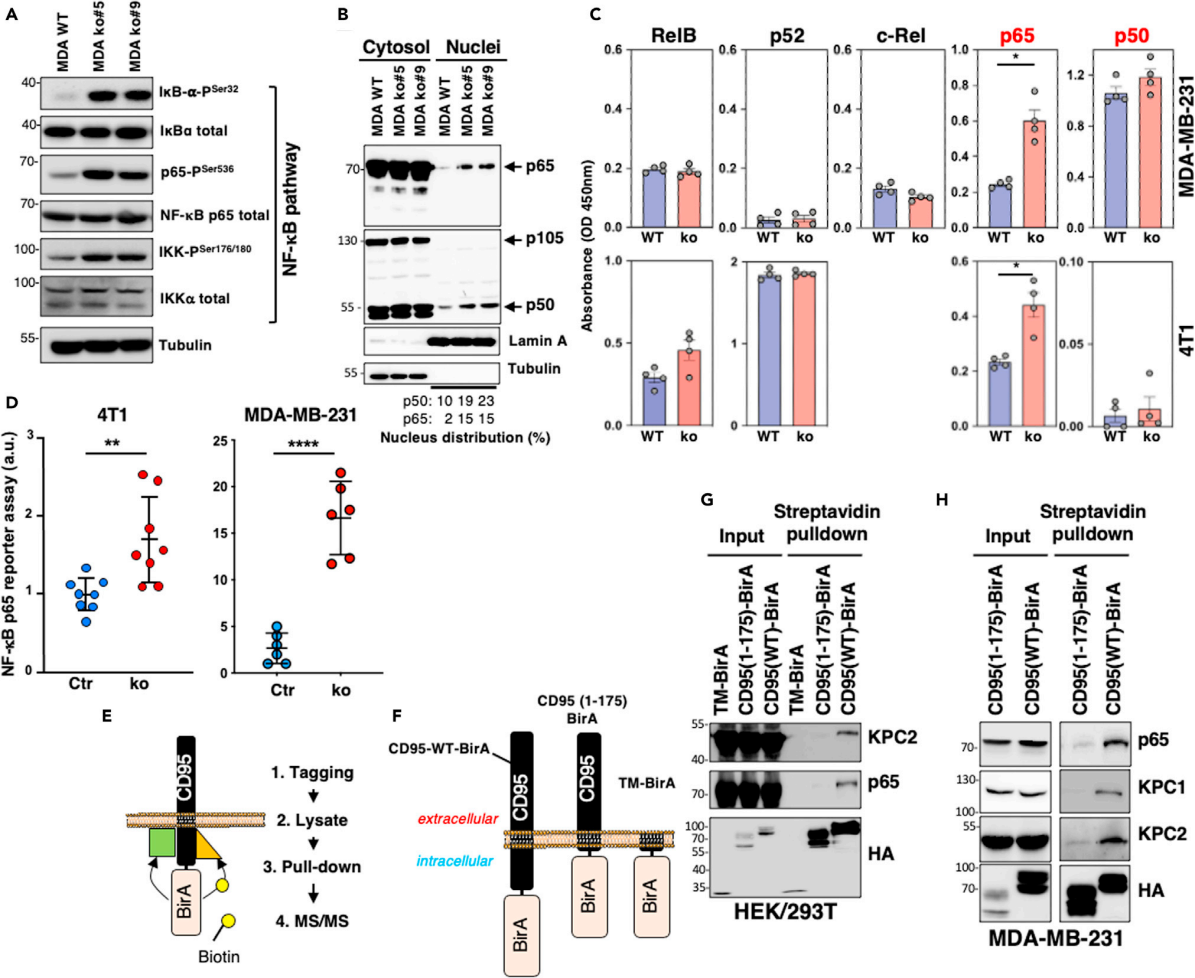
KPC2 and p65, two novel interactants of CD95 (A) The activation status of the NF-κB signaling pathway was analyzed in wild-type (WT) and CD95 knock-out (KO5 and KO9) MDA-MB-231 cells by immunoblotting with the indicated antibodies. Tubulin immunoblot serves as a loading control. Images are representative of three independent experiments. (B) The presence of p65, p105, and p50 in the whole lysate (cytosol) or the nucleus fraction of wild-type and CD95 k.o. cells was evaluated by immunoblotting. Lamin A and tubulin serve as loading controls for nucleus and cytosolic fractions, respectively. Images are representative of three independent experiments. (C) Nuclear extracts from WT and CD95 k.o. cells were subjected to the indicated ELISA to quantify activation of NF-kB. Mouse c-Rel DNA binding cannot be assessed with this kit. Mean ± SEM (n = 3), p values were calculated using nonparametric Mann-Whitney test. (D) RelA activity was measured in indicated tumor cells using luciferase reporter assay (n = 6–8). ∗∗ and ∗∗∗∗ stand for p < 0.001 and p < 0.00001, respectively, using unpaired and nonparametric Mann-Whitney t test. (E) Schematic representation of the BioID experiment. (F) Schematic representation of BirA-fused CD95 constructs. (G and H) BioID assay was conducted in CD95 k.o. HEK/293T cells (G) and MDA-MB-231 cells (H) reconstituted with indicated constructs and after streptavidin pull-down indicated immunoblotting was performed. Images are representative of three independent experiments.

The differential transcriptomic signatures and the associated GSEA suggested that the NF-κB signaling pathway could be induced when CD95 expression was eliminated in TNBC cells. In agreement with this observation, deletion of CD95 in MDA-MB-231 cells led to a potent activation of the NF-κB signaling pathway as monitored by the phosphorylation of IKKα/β at Ser176/177 and Ser180/181, IκBα at Ser32, and p65 at Ser536 (Sakurai et al., 1999) (all hallmarks of NF-κB activation) (Figure 2A). The five members of the NF-κB family including RelA (p65), RelB, c-Rel, NF-κB1 (p105), and NF-κB2 (p100) share a conserved Rel homology domain (RHD) responsible for DNA binding. However, only p65, RelB, and c-Rel contain a transactivation domain in their C-terminal regions. Because p50 is devoid of transactivation domains, nuclear accumulation of p50/p50 homodimers is considered as transcriptionally repressing the NF-κB response (Grundstrom et al., 2004; Kang et al., 1992; Kravtsova-Ivantsiv et al., 2015; Udalova et al., 2000; Zhong et al., 2002). Although the quantity of p50 was slightly increased in nuclei of CD95 k.o. cells when compared with parental MDA-MB-231 cells (Figures 2B and 2C), this was less pronounced when compared with the nuclear accumulation of p65 (Figures 2B and 2C). In agreement with the accumulation of an active p50/p65 heterodimer in nuclei of CD95 k.o. TNBC cells, an NF-κB reporter assay confirmed that CD95 loss in human (MDA-MB-231) and mouse (4T1) TNBC cells resulted in the induction of this signaling pathway (Figure 2D).

These findings raised the question of how CD95 expression negatively regulated this form of NF-κB signaling and how its loss in TNBC cells triggered this pro-inflammatory program. To characterize in an unbiased fashion the CD95 interactome in unstimulated cells and identify factors that might contribute to the NF-κB regulation, we conducted a proteomic analysis using the proximity-dependent biotin identification (BioID) method (Roux et al., 2012). Briefly, the activated *Escherichia coli* BirA biotin ligase (mutant R118G) was fused to CD95 (Figure 2E). This enzyme has the ability to biotinylate surrounding proteins located within a radius of 10 nm. Three constructs consisting of wild-type CD95, a CD95 devoid of its intracellular region (CD95(1–175)-BirA), and the transmembrane domain of CD95 (TM-BirA) were fused to BirA (Figure 2F) and transiently expressed in CD95 k.o. HEK/293T cells. By fusing CD95 to the BirA domain, even proteins interacting weakly and transiently with the receptor can be biotinylated in an irreversible fashion and thereby detected by mass spectrometry analysis (MS). Cells were lysed, and biotin-conjugated proteins were next precipitated using streptavidin beads and identified by MS/MS. Among the 198 proteins selectively tagged by BirA and associated with the wild-type CD95 construct were caspase-8 and CD95 validating the method to characterize the CD95 interactome (Table S5). Of note, some biotinylated factors corresponded to inhibitors of the NF-κB pathway including COMMD7 (Esposito et al., 2016), KPC2 (Kravtsova-Ivantsiv et al., 2015), or TRAF-D1 (Sanada et al., 2008) (Table S5). In addition, the transcription factor RelA/p65 was detected (Table S5). Pull-down experiments confirmed that KPC2 (also known as UBAC1) and p65 were associated with wild-type CD95, and these interactions were lost with CD95(1–175) devoid of its intracellular region (Figure 2G).

NF-κB1 (p105) is synthesized as a large precursor, which is processed to generate the NF-κB subunit p50. KIP1 ubiquitination-promoting complex (KPC) is a ubiquitin ligase responsible for the partial degradation of p105 into p50 (Kravtsova-Ivantsiv et al., 2015). This complex consists of KPC2, which stabilizes KPC1 (Hara et al., 2005), a RING-finger protein serving as the ligase (Kravtsova-Ivantsiv et al., 2015). We next evaluated whether similarly to HEK/293T cells, CD95 was associated with p65 and KPC2 in the TNBC cell line MDA-MB-231. CD95 k.o. cells were reconstituted with a wild-type CD95 or a truncated receptor devoid of its intracellular region fused to BirA (Figure 2H). A pull-down assay confirmed that KPC2 and p65 were close to CD95 in unstimulated TNBC cells (Figure 2H). In addition, KPC1 was also detected in the biotinylated complex. On the other hand, TRAF-D1 and COMMD7 were not detected in HEK/293T and MDA-MB-231 cells, indicating that either these factors were present in the unstimulated CD95 complex but to a lesser extent when compared with KPC2 and p65 or that these factors corresponded to false-positive hits. Overall, these findings suggested that the CD95 expression in TNBC cells regulates NF-κB signaling through a KPC-dependent mechanism.

### KPC2 binds the C-terminal region of CD95

**Figure 3:**
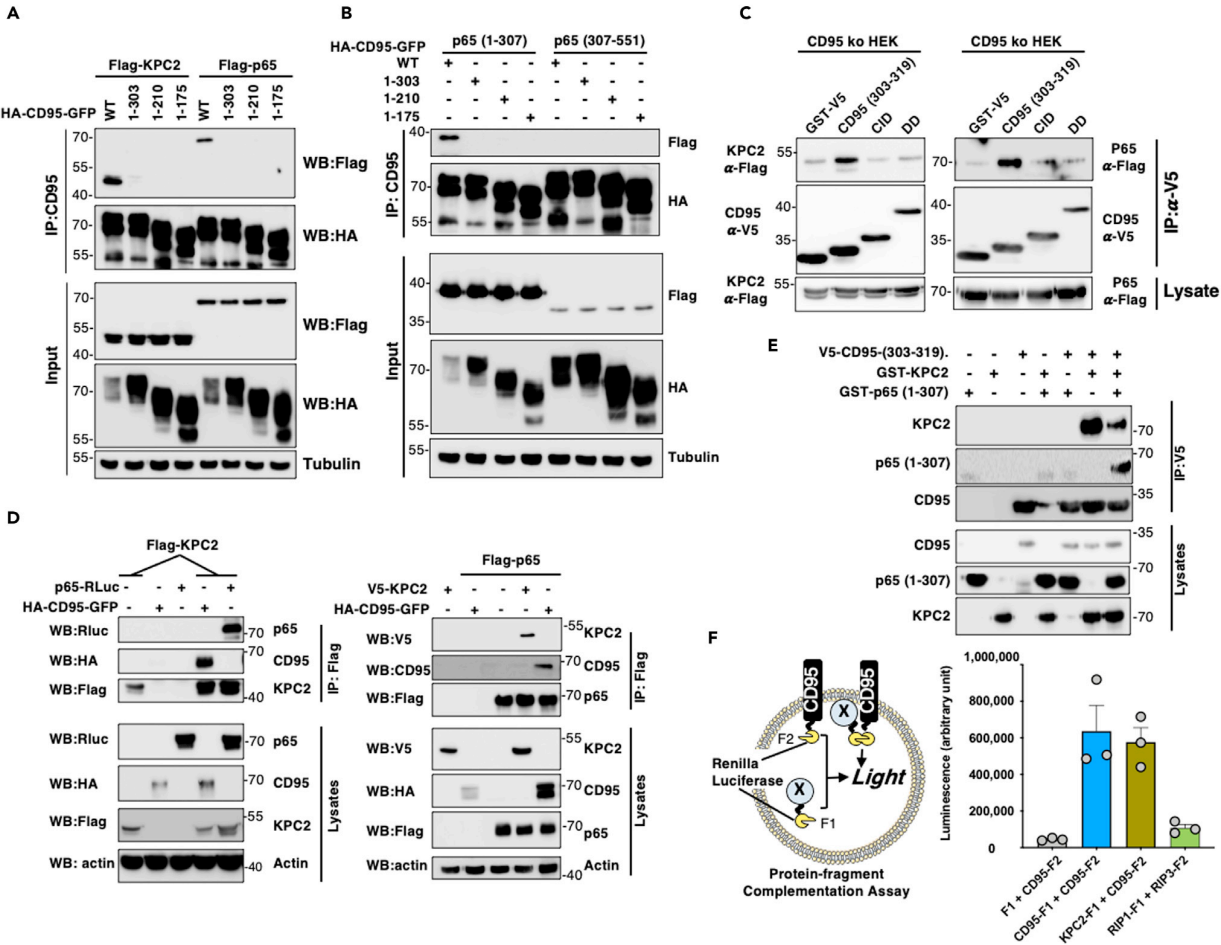
KPC2 binds the C-terminal domain of CD95 and it is an adaptor for p65 and KPC1 (A) CD95 k.o. HEK/293T cells were transfected with Flag-KPC2 or Flag-p65 and indicated HA-tagged CD95-GFP constructs. After 24 h, cells were lysed, HA immunoprecipitations were performed, and the immune complex was resolved by SDS-PAGE. Indicated immunoblotting was realized. Images are representative of three independent experiments. (B) CD95 k.o. HEK/293T cells were co-transfected with Flag-tagged N-term (1–307) or C-terminal (307–551) p65 and with indicated HA-CD95-GFP constructs. Twenty-four hours after transfection, cells were lyzed and HA immunoprecipitations were performed. Immune complex was resolved by SDS-PAGE and indicated immunoblotting was conducted. Images are representative of three independent experiments. (C) Indicated GST-V5-CD95 domains (100 ng) were produced in E.*coli*. and mixed with lysate (1 mg) of HEK/293T cell transfected either Flag-KPC2 or Flag-p65. CD95 domains were immunoprecipitated using anti-V5 mAb and the presence of KPC2 or p65 was evaluated by immunoblots. Images are representative of three independent experiments. (D) CD95 k.o. HEK/293T cells were co-transfected with Flag-KPC2 (left panels) or Flag-p65 (right panels) and HA-CD95 WT in the presence of p65-Renilla luciferase (domain F1, left panels) or V5-KPC2 (right panels). After 24 h, Flag immunoprecipitations were performed and the immune complex was resolved by SDS-PAGE. Indicated immunoblotting was performed. Images are representative of three independent experiments. (E) GST-KPC2, GST-p65 (1–307), and GST-V5 CD95 (303–319) were produced in *E*. *coli* and purified. Indicated proteins (100 ng) were mixed for 1 h, and V5 immunoprecipitation was performed. The immune complex was resolved by SDS-PAGE and indicated western blots were performed. Images are representative of three independent experiments. (F) Protein-fragment complementation assay (PCA). HEK/293T cells were co-transfected with CD95 fused to the C-term region of Renilla luciferase (F2), and the N-term part of Renilla luciferase (F1) conjugated with the indicated proteins and luciferase activity was measured. RIP1/RIP3 interaction served as a positive control. Data represent mean ± SD (n = 3).

We next wondered how CD95 interacted with KPC2 and p65. Although immunoprecipitation of wild-type CD95 confirmed its association with both KPC2 and p65 (Figures 3A and 3B), the CD95 construct consisting in the amino acid residues 1 to 303 and thereby deleted of its C-terminal region failed to interact with KPC2 (Figure 3A) or p65 (Figure 3B), suggesting that p65 and KPC2 were recruited via the very C-terminal end of CD95 (amino acid residues 303 to 319.) Interestingly, only the amino terminal region of p65 (amino acid residues 1 to 307) encompassing its nuclear localization sequence (NLS) interacted with CD95 (Figure 3B), suggesting that CD95 could prevent nuclear-localization of p65 by masking its NLS similarly to inhibitor of kappa B (IκB) (Beg et al., 1992). To confirm that p65 and KPC2 required the C-terminal region of CD95 to be recruited, we synthesized in bacteria and purified the different CD95 domains including CID, DD, and C-term fused to glutathione S-transferase (GST) and incubated them with lysates of CD95 k.o. HEK/293T cells transfected with KPC2 or p65. As shown in Figure 3C, only pull-down of the C-terminal region of CD95 revealed the formation of a complex containing p65 and KPC2.

Immunoprecipitation of KPC2 in CD95 k.o. HEK cells transfected with wild-type CD95 confirmed the existence of a complex containing both CD95 and p65, and similarly, p65 precipitation showed the presence of CD95 and KPC2 in the immune complex (Figure 3D). Next, we evaluated whether the KPC2 and p65 interacted directly with CD95. Using GST pull-down assay in a cell-free system, we observed that although GST-CD95 directly bound KPC2, the intracellular region of the receptor failed to pull down p65. Interestingly, the CD95 C-terminal domain (amino acid residues 303–319) recruited p65 only when KPC2 was added to the CD95/p65 mix (Figure 3E), indicating that KPC2 acts as an adaptor between CD95 and p65.

To confirm that the KPC2/CD95 interaction was direct, we next developed a protein-fragment complementation assay (PCA) (Stefan et al., 2007), in which the Renilla luciferase enzyme was split into two fragments (F1 and F2) and fused to different proteins. When the proteins interact, their interaction favors luciferase refolding and the subsequent recovery of its enzymatic activity (Figure 3F, left). This method confirmed the direct interaction between full-length KPC2 and the intracellular region of CD95 (Figure 3F, right). In addition, co-transfection of the entire intracellular region (a.a. 175–319) or the C-terminal region (a.a. 303–319) of CD95 fused to F1 luciferase with full-length KPC2-F2 was able to refold the Renilla luciferase and allow light emission, whereas DD (a.a. 211–303) or CID (a.a. 175–210) CD95-F1 constructs failed to do it (Figure S2A). Finally, we developed a PCA competitive assay (Figure S2B) and established that the CD95/KPC2 interaction was only disrupted by the overexpression of the whole intracellular region or the C-terminal region (303–319 residues) of CD95 (Figures S2B and S2C). Overall, these findings indicated that KPC2 is a previously unrecognized binding partner of CD95, interacting with its C-terminal region.

### CD95 complex ubiquitinates p105 through KPC1/KPC2

**Figure 4:**
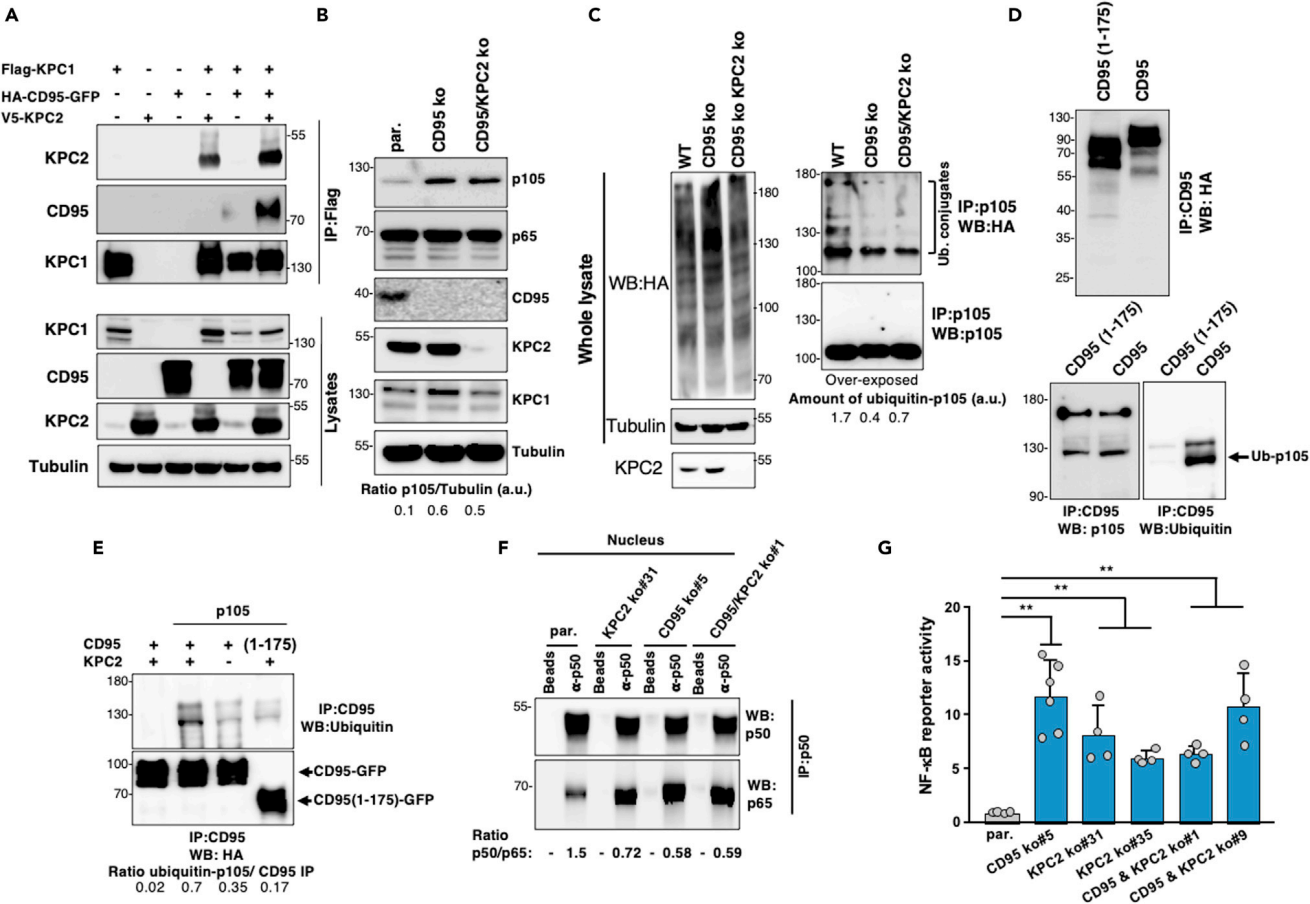
CD95 loss promotes the partial degradation of p105 and the nucleus accumulation of p50 homodimers (A) CD95 k.o. HEK/293T cells were co-transfected with Flag-KPC1, HA-CD95-GFP, and V5-KPC2 as indicated. After 24 h, KPC1 was immunoprecipitated, and the immune complex was resolved by SDS-PAGE, and indicated western blots were realized. Images are representative of three independent experiments. (B) CD95 and KPC2 were knocked out in HEK/293T cells using CRISPR/Cas9, and the expression level of the KPC1/KPC2 substrate p105 was analyzed by immunoblot. Images are representative of three independent experiments. (C) CD95 and CD95/KPC2 k.o. HEK/293T cells were co-transfected with cDNAs encoding for p105 and HA-Ubiquitin. After 24 h, the proteasome inhibitor MG132 was added for 3 h, cells were lysed, and p105 was immunoprecipitated. Total lysate (left panels) and p105 immunoprecipitation (right panels) are depicted. Following SDS-PAGE, indicated immunoblots were performed. Images are representative of three independent experiments. (D) CD95 k.o. HEK/293T cells were transfected with full length or intracellular-truncated (1–175) CD95 fused to GFP, and CD95 complexes were immunoprecipitated and subjected to an *in vitro* ubiquitin conjugation assay in a reconstituted cell-free system in the presence of recombinant human p105 as described in STAR Methods. Then proteins were resolved by SDS-PAGE, and indicated immunoblots were performed. Data are representative of three independent experiments. (E) CD95/KPC2 double k.o. HEK/293T cells were transfected with full length or intracellular-truncated (1–175) CD95-GFP and KPC2. Then, cells were lysed, and CD95 was immunoprecipitated. The immune complexes were subjected to an *in vitro* ubiquitin conjugation assay as depicted in D, and next, proteins were resolved by SDS-PAGE, and indicated immunoblots were performed. Data are representative of three independent experiments. (F) Nuclei of parental (par.), CD95 k.o. and KPC2/CD95 k.o. MDA-MB-231 cells were isolated, and p50 was immunoprecipitated. The immune complex was resolved by SDS-PAGE, and co-association with p65 was evaluated by immunoblotting. Data are representative of three independent experiments. (G) RelA (p65) activity was measured in parental, CD95 k.o. and KPC2/CD95 k.o. MDA-MB-231 cells using luciferase reporter assay (n = 4–6). ∗∗ stands for p < 0.001 using unpaired and nonparametric Mann-Whitney t test.

In the Kip1 ubiquitination-promoting complex (KPC), KPC1 polyubiquitinates the precursor p105, leading to its partial degradation giving rise to the p50 subunit (Kravtsova-Ivantsiv et al., 2015). KPC1 was detected in the CD95 complex precipitated in MDA-MB-231 cells (Figure 2H). Similarly to CD95 k.o. HEK/293T cells, co-immunoprecipitation experiments confirmed that KPC1 was associated with KPC2 (Figure 4A). The immunoprecipitation of KPC1 also revealed a faint quantity of CD95, probably due to the low endogenous level of KPC2 present in HEK/293T cells (Figure 4A). Indeed, this amount dramatically increased when KPC2 was co-transfected (Figure 4A), suggesting that similar to p65, KPC1 was indirectly recruited to CD95 via its interaction with KPC2. Strikingly, deletion of CD95 in HEK cells resulted in an accumulation of p105 when compared with parental cells (Figure 4B) without affecting the expression level of p65 (Figure 4B). In agreement with its KPC1 stabilizer role (Kravtsova-Ivantsiv and Ciechanover, 2015), KPC2 deletion in CD95 k.o. cells reduced the expression level of KPC1 (Figure 4B). To evaluate the interplay between KPC2 and CD95 in the p105 ubiquitination and the downstream NF-κB activation, we took advantage of that transient, and multiple transfections were more efficient in HEK cells as compared with MDA-MB-231 cells. Interestingly, double knock-out of CD95 and KPC2 did not translate into a more pronounced accumulation of p105 when compared with CD95 k.o. cells (Figure 4B), suggesting that the KPC-mediated regulation of p105 expression mainly relies on CD95 expression. Because p105 expression relied on its ubiquitination, we evaluated whether CD95 expression promoted p105 ubiquitination. To address this question, we immunoprecipitated p105 in CD95 k.o. or CD95/KPC2 k.o. cells co-transfected with Flag-p105 and HA-tagged ubiquitin (Figure 4C). The immunoprecipitation of p105 did not reveal an upper band in the anti-p105 immunoblot, suggesting that a minor part of p105 underwent ubiquitination (Figure 4C). Nonetheless, HA staining confirmed that ubiquitin was added to p105 in wild-type cells, and this conjugation was dramatically reduced in CD95 k.o. cells (Figure 4C). Interestingly, the combined deletion of KPC2 and CD95 did not enhance this reduction (Figure 4C), supporting that KPC2 was mandatory for the CD95-driven ubiquitination of p105.

To establish that CD95 promoted p105 ubiquitination, we analyzed p105 ubiquitin conjugation in a reconstituted cell-free system from immunoprecipitation of full-length CD95 or CD95(1–175) constructs transfected in CD95 k.o. HEK/293T cells (Figure 4D). p105 was efficiently ubiquitinated when incubated with the full-length CD95 immune complex (Figure 4D), whereas immunoprecipitation of a CD95 devoid of its intracellular region (CD95(1–175)) failed to add ubiquitin moieties to p105 (Figure 4D) confirming that in unstimulated cells, a CD95 complex efficiently ubiquitinates p105. To confirm that KPC2 was critical for the CD95-dependent p105 ubiquitination, similar experiments were carried out using CD95/KPC2 double k.o. HEK/293T cells co-transfected with full-length CD95 or CD95(1–175) construct and KPC2 (Figure 4E). Although the same quantity of CD95 was immunoprecipitated, the magnitude of p105 ubiquitination significantly dropped in the CD95-containing immune complex devoid of KPC2 (Figure 4E), validating that CD95/KPC2 interaction caused p105 ubiquitination.

Because p50 does not contain a transactivation domain (TAD) and its homodimeric form mostly acts as a transcriptional repressor (Christian et al., 2016), we wondered whether the CD95-driven ubiquitination of p105 could favor the accumulation of p50 homodimers in the nucleus at the expense of the transcriptional activator p50/p65. To tackle this question, we generated KPC2 k.o. and double k.o. MDA-MB-231 cells from the CD95 k.o. MDA-MB-231 clone 5 and analyzed the ratio of p50 and p65 in nuclei of these cells (Figure 4F). Immunoprecipitation of p50 revealed an NF-κB complex enriched in p50 in nuclei of parental TNBC cells as compared with those isolated from CD95 k.o., KPC2 k.o., and double k.o. counterparts (Figure 4F). Densitometry analysis revealed that the p50/p65 ratio in parental cells dropped from 1.5 to 0.72 in KPC2 k.o. cells and 0.58 and 0.59 in CD95 k.o. and CD95/KPC2 double k.o. TNBC cells, respectively. This increase in the p50/p65 complex occurring in the nuclei of CD95 k.o., KPC2 k.o., and CD95/KPC2 double k.o. MDA-MB-231 cells at the expense of the inhibitor p50 homodimer suggested that the NF-κB activity could be augmented in these cells. To evaluate this, an NF-κB reporter assay was performed in these cells, confirming that elimination of KPC2 or CD95 in TNBC cells resulted in a robust NF-κB response (Figure 4G). This increased NF-κB signaling was similar to that observed in CD95/KPC2-double KO cells (Figure 4G), again supporting the idea that these two factors shared the same signaling pathway to inhibit the NF-κB transcriptional activity. Finally, we evaluated whether the secretion of the five pro-inflammatory cytokines was affected in KPC2 k.o. and CD95/KPC2 double k.o. TNBC cells (Figure S2D). ELISAs confirmed that similarly to CD95 k.o. cells, elimination of KPC2 increased the secretion of these cytokines (Figure S2D). Moreover, the increased secretion of these molecules was not amplified in CD95/KPC2 double k.o. TNBC cells when compared with unique k.o. cells, supporting that CD95 and KPC2 share a common signaling pathway to inhibit the expression of these pro-inflammatory cytokines.

Overall, these findings demonstrate that KPC2 binding to CD95 not only contributes to the ubiquitination and partial degradation of p105 but also sequesters its transcriptionally active partner p65 to the plasma membrane. Deletion of CD95 therefore promotes the activation of the NF-κB pathway that causes induction of inflammatory chemo- and cytokines.

## Discussion

Stimulation of CD95 on signaling competent but apoptosis-resistant cancer cells results in the activation of multiple nonapoptotic signaling pathways that include NF-κB that drives tumor promotion (Legembre et al., 2004). We now show that the role of CD95 and its link to the NF-κB signaling pathway on TNBC cancer cells, which are virtually signaling incompetent, appears to be different. Our study suggests that in unstimulated cancer cells, CD95 forms a complex comprised of KPC2/KPC1 and p65 that favor the generation of the p50/p50 homodimer at the expense of its pro-inflammatory p50/p65 heterodimer. Although the p50 homodimer may act as a transcriptional repressor because it lacks a transactivation domain (May and Ghosh, 1997), it can also interact with different transcriptional modulators, such as Bcl-3 (Fujita et al., 1993), p300 (Deng and Wu, 2003), or HMGA1/2 (Perrella et al., 1999). Consequently, an imbalance of the p50 expression will shift the composition of NF-κB dimers, resulting in a different cellular response to environmental stresses.

Our study identifies a novel mechanism that might account for the anti-tumor activity of NK cells observed in TNBCs, after deletion of CD95 (Qadir et al., 2021). It involves sequestering a fraction of p65 at the plasma membrane and simultaneously promotes partial degradation of p105 to form p50, favoring the formation of p50 homodimers at the expense of the pro-inflammatory p50/p65 complex (Kravtsova-Ivantsiv et al., 2015). Combined with our recent study in TNBCs (Qadir et al., 2021), this work highlights a complex picture: although CD95 loss in tumor cells in TNBC patients can curtail the anti-tumor activity of CD95L-expressing T- and NK cells, it also induces an inflammatory signature, resulting in a more pronounced NK-mediated anti-tumor response (Qadir et al., 2021). Besides NK cells, the expression of pro-inflammatory chemokines including CSF1, CXCL1, and CXCL2 may also favor the trafficking of macrophages as observed in our animal models (Qadir et al., 2021).

A comprehensive genomic analysis of breast cancer (Cancer Genome Atlas, 2012) cells previously identified the transcription factors, FOXM1 and c-myc, which are hyperactivated in TNBCs as factors to contribute to the downregulation of the CD95 expression (Penke et al., 2018; Zhang et al., 2017). Furthermore, breast cancers in general upregulate the PI3K signaling pathway through the expression of constitutive active or amplification of PIK3CA and deletion of PTEN or INPP4B or Akt mutations (Nik-Zainal et al., 2016). This oncogenic signaling pathway activates the transcription repressor YY1 shown to be involved in the downregulation of CD95 mRNA expression (Garban and Bonavida, 2001). Interestingly, although these transcription regulations might explain the reduction of CD95 observed in many tumors, the estrogen receptor-negative TNBC cells maintain a higher level of CD95 as compared with other breast tumors (Blok et al., 2017). Although the classical tumor evasion hypothesis associated with the CD95 loss in tumor cells remains valid, this immune selection might be counterbalanced by the induction of an NF-κB-driven pro-inflammatory response, which could activate the anti-tumor activity of NK cells that we observed in TNBCs (Qadir et al., 2021). We also observed that soluble CD95L is increased in TNBC women and triggers a PI3K signaling pathway, promoting the migration of the cancer cells (Malleter et al., 2013). Overall, it could be interesting to evaluate how the receptor alone or in the presence of its apoptotic (i.e., membrane-bound CD95L) and nonapoptotic (i.e., metalloprotease-cleaved CD95L) ligand will contribute to the carcinogenic process and to decipher the molecular mechanisms responsible for switching from one signaling complex to another one.

Importantly, our work raises the question of whether the CD95-mediated inhibition of NF-kB is a mechanism only seen in oncogenesis or whether it also plays a role in more physiological processes. For instance, B lymphocytes entering the germinal center (GC) are phenotypically characterized by the overexpression of CD95, an observation that has been associated to the elimination of the low antigen affinity B lymphocytes (Hennino et al., 2001). Based on our study, we hypothesize that CD95 overexpression in GC B cells could exert a different function by controlling B cell maturation. NF-κB promotes the expression of activation-induced cytidine deaminase (AID) (Endo et al., 2007), an enzyme responsible for the GC B cell maturation by initiating class switch DNA recombination (CSR) and somatic hypermutation (SHM). Interestingly, NF-κB-dependent AID overexpression results in an auto-immune disorder (Gan et al., 2020), recapitulating features observed in mice with a conditional loss of CD95 in B cells (Hao et al., 2008). The transcription repressor BCL6 is involved in the development of germinal centers (GCs) and in lymphomagenesis (8). BCL6 inhibits the expression of DNA damage checkpoint genes, to promote B-cell proliferation during SHM and CSR of immunoglobulin genes (Phan and Dalla-Favera, 2004). Although in GC B cells CD40 stimulation has been reported to stimulate NF-κB, which in turn represses BCL6 (Saito et al., 2007), we hypothesize that CD95 overexpression in GC B cells might inhibit the NF-κB response in these cells and thereby contribute to the affinity maturation of antibodies. Among 189 mutations annotated in the 335-length CD95 amino acid sequence, we found a unique mutation in the C-terminal region of CD95 (Tauzin et al., 2012), and this mutation was detected in Reed-Sternberg (H/RS) cells, derived from GC B cells (Muschen et al., 2000). Interestingly, the H/RS patient harbored a monoallelic somatic mutation at E307 in the C-terminal region of CD95, suggesting that similar to mutations in the CD95 DD, C-terminal mutations might exert a dominant effect. Whether this occurs through the regulation of the NF-κB signaling remains to be elucidated.

### Limitations of the study

Combined with the study showing that CD95 expression in TNBC cells exerts an inhibitory effect on the anti-tumor activity of NK cells, this work raises the question of whether the CD95-dependent p105 ubiquitination and the downstream inhibition of NF-κB are responsible for the general increase in trafficking of macrophages, T cells (CD4+ and CD8+), and NK cells observed in CD95 k.o. TNBCs when compared with parental tumors or whether this mechanism accounts for a more subtle and selective effect on certain immune cells. To decipher the set of chemo- and cytokines whose regulation by CD95 expression or CD95 loss contributes to this immune effect will be of interest too. These questions will be investigated in the future using appropriate mouse cell lines and animal models.

## STAR★Methods

### Key resources table

**Table:** 

REAGENT or RESOURCE	SOURCE	IDENTIFIER
**Antibodies**		

Lamin A antibody	Santa Cruz	Cat.# sc-20680; RRID: AB_648148
KPC1 antibody	Santa Cruz	Cat.# sc-101122; RRID: AB_2182272
KPC2 antibody	Abcam	Cat.# ab177519
Anti-Flag (clone M2) antibody	Sigma Aldrich	Cat.# F1804; RRID: AB_262044
Anti-β Actin (clone AC-74) antibody	Sigma Aldrich	Cat.#A5316; RRID:AB_476743
Anti-α Tubulin (clone DM1A) antibody	Sigma Aldrich	Cat.# T6199; RRID:AB_477583
Anti-HA.11 antibody	Biolegend	Cat.# 901513; RRID:AB_2565335
Anti-tag V5 antibody	ThermoFisher.	Cat.# R960-25; RRID:AB_2556564
Anti-tag GST antibody	ThermoFisher	Cat.# CAB4169; RRID:AB_10709998
Anti-phospho-IκBα Ser32 antibody	Cell Signaling Technology	Cat.# 2859; RRID:AB_561111
Anti-I**κ**Bα antibody	Cell Signaling Technology	Cat.# 4814; RRID:AB_390781
Anti-phospho-IKK Ser176/177 antibody	Cell Signaling Technology	Cat.# 2078; RRID:AB_2079379
Anti-IKKα antibody	Cell Signaling Technology	Cat.#11930; RRID:AB_2687618
Anti-phospho-p65 Ser536 antibody	Cell Signaling Technology	Cat.# 3033; RRID:AB_331284
Anti-p65 antibody	Cell Signaling Technology	Cat.# 8242; RRID:AB_10859369
Anti-p105/p50 antibody	Cell Signaling Technology	Cat.#12540; RRID:AB_2687614
Anti-p27 antibody	Cell Signaling Technology	Cat.#3686; RRID:AB_2077850
Anti-CD95 mAb	Cell Signaling Technology	Cat.# 4233; RRID:AB_2100359
Anti-CD95 mAb (clone APO1-3)	Enzo	Cat.#ALX-805-020-C100; RRID:AB_2051388

**Bacterial andvirusstrains**		

BL21 *E.coli* strain.	New England Biolabs	Cat.#C2530H

**Chemicals, peptides, and recombinant proteins**		

recombinant human p105	Novus Biologicals	Cat.#H00004790
ubiquitin activating enzyme E1	Enzo Life Sciences	Cat.#BML-UW9410-0050
E2 UbcH5c	Enzo Life Sciences	Cat.#BML-UW9070-0100
Biotinylated Ubiquitin	Enzo Life Sciences	Cat.#BML-UW8705-0100
RPMI 1640 medium	Fisher Scientific	Cat.# 10040CM
Fetal bovine serum (FBS)	Sigma-Aldrich	Cat.# 14009C
L-glutamine	Fisher Scientific	Cat.# 25-005CI
Penicillin/Streptomycin	Fisher Scientific	Cat.# 30-002-CI
Propidium iodide	Sigma-Aldrich	Cat.# P4864
Bovine serum albumin	Sigma-Aldrich	Cat.# A7906
Puromycin	Sigma-Aldrich	Cat.# P9620
MG132	Sigma-Aldrich	Cat.# M7449
Biotin	Sigma-Aldrich	Cat.# B4639
G418	Sigma-Aldrich	Cat.# G8168
N-Ethylmaleimide (NEM)	Pierce	Cat.# 23030
Polybrene	Sigma-Aldrich	Cat.# H9268
Lipofectamine 3000	Thermo Fisher Scientific	Cat.# L3000015
Trypan blue solution	Lonza	Cat.# 17-942-E
Normal buffered formalin	VWR	Cat.# 16004-128
DNAse Set	Qiagen	Cat.# 79254
ECL revelblot	Ozyme	Cat.# OZYB001-5000
isopropyl b-D-1-thiogalactopyranoside (IPTG)	Sigma-Aldrich	Cat.#I5502
phenylmethylsulfonyl fluoride (PMSF)	Sigma-Aldrich	Cat.#11359061001
Inorganic pyrophosphatase	Sigma Aldrich	Cat.#I1643
Coomassie Brilliant Blue		
Coelenterazine-h	Promega	Cat.#S2011

**Critical commercial assays**		

NucleoSpin RNA Kit	Macherey-Nagel	Cat.#740955.50
high-capacity cDNA reverse transcription kit	Thermo Fisher Scientific	Cat.#4368814
SYBR Green PCR Master Mix	Applied Biosystems	Cat.#A46109
Nuclear Extract kit	Active Motif	Cat.#78833
TransAM NF-kB Family kit	Active Motif	Cat.#43296
Proteome Profiler Human XL Cytokine array	R&D Systems	Cat.#ARY022B
EnGen sgRNA Synthesis kit	NEB	Cat.#E3322S
RNA clean and concentrator	Zymo Research	Cat.#R1013
EnGen Cas9 NLS protein	NEB	Cat.#M0646T
Quantikine ELISA kits human GM-CSF (CSF2)	R&D Systems	Cat.#DGM00
Quantikine ELISA kits human M-CSF (CSF1)	R&D Systems	Cat.#DMC00B
Quantikine ELISA kits human CXCL1	R&D Systems	Cat.#DGR00B
Quantikine ELISA kits human IL1α	R&D Systems	Cat.# DLA50
Quantikine ELISA kits human IL1β	R&D Systems	Cat.# DLB50
Glutathione-Sepharose affinity columns	Sigma Aldrich	Cat.# GE17-5130
Anti-V5-agarose beads	Sigma Aldrich	Cat.#A7345
Anti-Flag M2 beads	Sigma Aldrich	Cat.#M8823
Protein A magnetic beads	Life Technologies	Cat.#10002D
High-Capacity Streptavidin Agarose beads	Pierce	Cat.#20357
Luciferase cell culture lysis buffer	Promega	Cat.#E1531
Luciferase assay system	Promega	Cat.#E1500

**Deposited data**		

RNAseq data of the combined analysis of 4T1 and MDA-MB-231 wt and CD95 k.o cell lines	Legembre Lab	GEO: GSE172215
BirA data have been deposited in ProteomeXchange Consortium via the PRIDE partner repository	Legembre Lab	PXD027196
Raw data from immunoblots were deposited on Mendeley at https://data.mendeley.com/datasets/c5fgwrsrbg/draft?a=c94017bb-ddc8-4abf-8208-290cea7ba959.	Legembre Lab	

**Experimental models: Cell lines**		

4T1 cell line	ATCC (Molsheim Cedex, France)	
MDA-MB-231 cell line	ATCC (Molsheim Cedex, France)	
HEK/293T	ATCC (Molsheim Cedex, France)	

**Oligonucleotides**		

All primers were from Eurogentec, Liège, Belgium IDT, Leuven, Belgium		
Human CSF1	AAGAGACCCTGCCCTACCTG	N/A
Human CSF1	AGCCGACCCTCACTTTCC	N/A
Human IL1B	ATGATGGCTTATTACAGTGGCAA	N/A
Human IL1B	GTCGGAGATTCGTAGCTGGA	N/A
Human CSF2	AGAAATGTTTGACCTCCAGGA	N/A
Human CSF2	TTGCACAGGAAGTTTCCG	N/A
Human IL1A	AACCAGTGCTGCTGAAGGA	N/A
Human IL1A	TTCTTAGTGCCGTGAGTTTCC	N/A
Human CXCL1	CGAAAAGATGCTGAACAGTGA	N/A
Human CXCL1	GCCTCTGCAGCTGTGTCTC	N/A
Complete description of primers in Table S6		

**Recombinant DNA**		

For all CD95 constructs, the numbering takes into consideration the subtraction of the 16 amino-acid signal peptide sequence.		
pcDNA3.1-PS-HA-hCD95(1-319)		Poissonnier et al. (2018), Nat Chem Biol.
pMCS-BioID2-HA	Addgene	Cat.#74224
PX459-V2	Addgene	Cat.#62988
HA-Ubiquitin	Addgene	Cat.#18712
pHAGE NF-κB-TA-LUC-UBC-GFP-W	Addgene	Cat.#49343
KPC2-hRluc-F[2]-pcDNA3.1(+)	Legembre Lab	N/A
p65-hRluc-F[2]-pcDNA3.1(+)	Legembre Lab	N/A
CD95(303-319)-hRluc-F[2]-pcDNA3.1(+)	Legembre Lab	N/A
CD95(303-319)-hRluc-F[1]-pcDNA3.1(+)	Legembre Lab	N/A
CD95(211-303)-hRluc-F[1]-pcDNA3.1(+)	Legembre Lab	N/A
CD95(175-210)-hRluc-F[1]-pcDNA3.1(+)	Legembre Lab	N/A
CD95(1-319)-hRluc-F[1]-pcDNA3.1(+)	Legembre Lab	N/A
pGEX4T1-KPC2	Legembre Lab	N/A
pGEX4T1-p65(1-307)	Legembre Lab	N/A
pGEX6P1-V5-CD95(175-319)	Legembre Lab	N/A
pGEX6P1-V5-CD95(211-303)	Legembre Lab	N/A
pGEX6P1-V5-CD95(175-210)	Legembre Lab	N/A
pGEX6P1-V5-CD95(303-319)	Legembre Lab	N/A
pLENTI6-Flag KPC1	Legembre Lab	N/A
pLENTI6-Flag KPC2	Legembre Lab	N/A
pLENTI6-Flag p65	Legembre Lab	N/A
pLENTI6-Flag p105	Legembre Lab	N/A
pLENTI6-Flag p65 (1-307)	Legembre Lab	N/A
pLENTI6-Flag p65 (307-551)	Legembre Lab	N/A
Complete description of vectors in Table S7		

**Software and algorithms**		

NovoExpress	Agilent	https://www.agilent.com/en/product/research-flow-cytometry/flow-cytometry-software/novocyte-novoexpress-software-1320805
Prism Software	Graphpad Software	https://www.graphpad.com/

**Other**		

Scanner LAS-4000 imager	Fujifilm	
Novocyte cytometer	ACEA Biosciences	
TECAN infinite 200 PRO plate reader	Tecan, Männedorf, Switzerland	

### Resource availability

#### Lead contact

Further information and requests for resources and reagents should be directed to and will be fulfilled by the lead contact, Patrick Legembre (Patrick.legembre@inserm.fr)

#### Materials availability

Plasmids and cell lines will be provided upon request.

### Experimental model and subject details

#### Cells lines

All cells were purchased from ATCC (Molsheim Cedex, France). The 4T1 cell line was cultured in RPMI supplemented with 8% heat-inactivated FCS (v/v) and 2 mM L-glutamine at 37°C and 5% CO_2_. HEK/293T and MDA-MB-231 cells were cultured at 37°C and 5% CO_2_ in DMEM supplemented with 8% heat-inactivated FCS and 2 mM L-glutamine.

### Method details

#### Reagents and antibodies

3-[4,5-dimethylthiazol-2-yl]-2,5-diphenyltetrazolium bromide (MTT) was purchased from Sigma-Aldrich (L’Isle-d’Abeau-Chesnes, France). Antibodies against phospho-IκBα Ser32 (#2859), IκBα (#4814), phospho-IKK Ser176/177 (#2078), IKKα (#11930), phospho-p65 Ser536 (#3033), p65 (#8242), p105/p50 (#12540), p27 (#3686) were purchased from Cell Signaling Technology. Antibodies against Lamin A (sc-20680) and KPC1 (sc-101122) were from Santa Cruz. KPC2 antibody was purchased from Abcam (ab177619) and antibodies against Flag (clone M2) and α-Tubulin (clone DM1A) were from Sigma Aldrich. Anti-HA.11 antibody was purchased from Biolegend and antibodies against V5 and GST tags were from ThermoFisher.

#### Plasmids

For all CD95 constructs, the numbering takes into consideration the subtraction of the 16 amino-acid signal peptide sequence. The vector PS-HA-hCD95(1-319) encodes the CD95 signal peptide followed by the human influenza hemagglutinin (HA) tag in frame with CD95 full length and GFP. Plasmids encoding the different CD95 mutants were previously described (Poissonnier et al., 2016). For all the constructs except the CD95(303-319) plasmids, we amplified the ORF genes by PCR and cloned them using the Gibson Assembly protocol (NEB). For the CD95 (303-319) plasmids, we directly ligated duplexed primers into the plasmid. The primers used for PCR are listed below. V5-tagged GST was generated by inserting between BamHI and EcoRI sites of pGEX6P1 the annealed V5 tag (GKPIPNPLLGLDST) using primers: GGTAAGCCTATCCCTAACCCTCTCCTCGGTCTCGATTCTACG and CGTAGAATCGAGACCGAGGAGAGGGTTAGGGATAGGCTTACC. For BioID experiments, wildtype or truncated CD95 sequence was extracted either from 5UTR-SP-HA-CD95 wt (1-319)-eGFP peGFP-N1 or 5’UTR-SP-HA-CD95 wt (1-175)-eGFP peGFP-N1 by NdeI and SmaI and inserted into the NdeI and AfeI sites of pMCS-BioID2-HA (Addgene #74224). The plasmid encoding the CD95 transmembrane domain fused in frame to the BirA was created by inserting the following annealed primers into the EcoRI and BamHI site of pMCS-BioID2-HA: AATTCTTGGGGTGGCTTTGTCTTCTTCTTTTGCCAATTCCACTAATTGTTTGGGTGG and GATCCCACCCAAACAATTAGTGGAATTGGCAAAAGAAGAAGACAAAGCCACCCCAAG. All primers were purchased from Eurogentec (Liège, Belgium).

The following sgRNA sequences were cloned within PX459-V2 plasmid and then transfected with lipofectamine 3000 in HEK/293T, 4T1 and MDA-MB231 cells to generate cell lines deficient for CD95 or CD95L according to manufacturer’s instructions. sgRNA sequences target human CD95, 5′CACCGAGGGCTCACCAGAGGTAGGA3’ (Fwd), 5′AAACTCCTACCTCTGGTGAGCCCTC3’ (Rev); mouse CD95 5′CACCGCTGCAGACATGCTGTGGATC3’ (Fwd), 5′AAACGATCCACAGCATGTCTGCAGC3’ (Rev); mouse CD95L 5′CACCGGTAATTCATGGGCTGCTGCA3’ (Fwd), 5′AAACTGCAGCAGCCCATGAATTACC 3’ (Rev). After transfection and puromycin selection for 48 h, genome-edited cells were cloned by limited dilution. To generate KPC2 knockout cells, sgRNA (CGGCGGCGGGATGTTCGTGC for KPC2) was synthetized *in vitro* using EnGen sgRNA Synthesis kit (NEB) and purified using RNA clean and concentrator (Zymo Research). Cells were then transfected with sgRNA and the EnGen Cas9 NLS protein (NEB) using Lipofectamine RNAiMAX and according to manufacturer’s instructions. After 48 h, cells were cloned by limiting dilution. Additional vectors are listed in Table S1.

#### Chemokine quantification

Cells (5.10^5^) were plated in 6-well plate and incubated for 4 days at 37°C. Media were then collected, filtered on 0.22 μm and immediately frozen. Cytokines level were measured using either the Proteome Profiler Human XL Cytokine array (R&D Systems) or Quantikine ELISA kits for dedicated cytokines (R&D Systems), according to manufacturer’s instructions.

#### Q-PCR

For qPCR analysis of cytokines expression, total RNA was isolated from cell lines using NucleoSpin RNA Kit (Macherey-Nagel) and subjected to Real-Time PCR using the high-capacity cDNA reverse transcription kit (Applied Biosystems, ThermoFisher Scientific). qPCR was performed using SYBR Green PCR Master Mix (Applied Biosystems). Results reported as fold change was calculated using the Δct method relative to the housekeeping gene GAPDH for Human or HPRT for mouse. Next, ΔΔct were assessed by comparing wildtype and KO cells. Primers purchased from Eurogentec (Angers, France) are listed in Table S2.

#### Ubiquitination

HEK/293T cells were transfected with plasmids encoding for Flag-p105 or Flag-p27 together with the plasmid HA-Ubiquitin (Addgene #18712). After 24 h, cells were treated for 3 h in the presence of 20 μM of proteasome inhibitor MG132, washed twice with PBS and lysed in IP buffer (50 mM Tris pH 7.4, 150 mM NaCl, 2 mM EDTA, 1% Triton X-100 and proteases inhibitor) supplemented with 5 mM of freshly added N-Ethylmaleimide (NEM). After sonication, lysates were clarified, and Flag-tagged protein were precipitated using anti-Flag M2 magnetic beads (Sigma Aldrich). After several washes in IP buffer, precipitated proteins were resolved by SDS-PAGE and immunoblotting was performed with the indicated antibodies.

#### NF-**κ**B ELISA

To quantify the level of NF-kB transcription factor activity, fractionation of MDA-MB-231 and 4T1 cells was first performed using the Nuclear Extract kit (Active Motif). The level of protein binding to DNA consensus sequence was then assessed by ELISA using the TransAM NF-kB Family kit (Active Motif) according to manufacturer’s instructions.

#### Subcellular fractionation

Cell lysis and immunoblot analysis were performed as described previously (Poissonnier et al., 2018). Proteins were visualized by enhanced chemiluminescence using ECL revelblot (Ozyme) and scanned with LAS-4000 imager (Fujifilm). Subcellular protein fractionation was performed using the REAP protocol as described (Suzuki et al., 2010). Briefly, cells were washed in PBS, and resuspended in lysis buffer (PBS, 0.1% NP-40). Lysate was centrifuged for 10 sec at 13,000 rpm and the supernatant was collected (cytoplasmic fraction). Pellet was then washed in lysis buffer, centrifuged for 10 sec at 13,000 rpm and the pellet was resuspended in lysis buffer and sonicated (nuclear fraction).

#### Protein production and pulldown assay

GST-V5 encoding wildtype and truncated CD95, GST-KPC2 and GST-p65 (1-307) constructs were transformed in BL21 *E.coli* strain. Bacteria were grown at 37°C in Luria Broth to an optical density of 0.8, and protein expression was induced with 0.5 mM isopropyl b-D-1-thiogalactopyranoside (IPTG) at 30°C for 4 hours. After centrifuging the cells, the pellets were resuspended in phosphate buffered saline (PBS) (137 mM NaCl, 2.7 mM KCl, 10 mM Na2HPO4, 2 mM KH2PO4, pH 7.4), containing 1 mM phenylmethylsulfonyl fluoride (PMSF), and 0.1 mg/ml lysozyme, and cells were lysed by sonication. Cell debris was removed by centrifugation, and the GST-fusion protein was purified using Glutathione-Sepharose affinity columns (GE Healthcare). The proteins were finally eluted in PBS containing 20 mM glutathione (pH 8) and the concentration and purity assessed by SDS-PAGE using Coomassie Brilliant Blue.

100 ng of CD95 constructs (GST-V5-CD95 (175-319), GST-V5-CD95 (175-210), GST-V5-CD95 (211-303) and GST-V5-CD95 (303-319)) and control GST-V5 were mixed with 100 ng of GST-KPC2 or GST-p65 (1-307) in IP Buffer (25 mM Hepes, 150 mM NaCl, 2 mM EDTA and 1% Triton X100 and protease inhibitors) for 1 h at room temperature. Anti-V5-agarose beads (Sigma Aldrich) were then added and incubated for 2 h at 4°C on rotating wheel. After 4x washes in IP buffer, the pulled-down complex was resolved by SDS-PAGE and analyzed by immunoblotting.

#### Immunoprecipitation

For classical immunoprecipitations, indicated cells were washed once in phosphate-buffered saline and lysed in IP buffer. Protein lysates were incubated overnight with 5 μL of Flag or V5 antibody-conjugated beads (Sigma-Aldrich) or with 1 μl of p50 antibody together with 20 μl of Protein A magnetic beads (Life Technologies). After 4 washes in IP buffer, the beads were resuspended in 2X Laemmli's buffer and subjected to immunoblotting analysis.

For immunoprecipitation of CD95 complexes, HEK/293T cells were transfected with Flag-KPC2 or Flag-p65. After 24 h, transfected cells were lysed in Hepes Buffer (Hepes 25 mM, NaCl 150 mM, NaF 2 mM, NaVO_4_ 1 mM, EGTA 2 mM). Cell lysates were incubated for 2 h at 4°C with 100 ng of the different GST-V5-CD95 constructs and then overnight at 4°C with 10 μl of Anti-V5 Agarose Beads (Sigma-Aldrich). After extensive washing in Hepes buffer, the precipitated complex was resolved by SDS-PAGE and immunoblotting was performed with the indicated antibodies.

#### BioId and proteomic analysis

HEK/293T cells were transfected with plasmids encoding either wild-type (CD95(WT)-BirA), CD95 devoid of its intracellular region (CD95(1-175)-BirA) or CD95 transmembrane domain (TM-BirA) fused in frame with BirA-R118G sequence. After 24 h, Biotin (50 μM) was incubated with cells for 16 hours. After 3 PBS washes, cells were scraped in lysis buffer (50 mM Tris pH 7.4, 500 mM NacL, 0,4% SDS, 1 mM DTT, 2% Triton X-100 and protease inhibitors), sonicated. Cell lysate was incubated with High-Capacity Streptavidin Agarose beads (Pierce) overnight at 4°C. Beads were washed once in 2% SDS, twice in modified RIPA buffer (50 mM Tris pH7.4, 150 mM NaCl, 1 mM EDTA, 0.1% SDS, 1% NP40), twice in TAP buffer (50 mM Hepes pH8, 150 mM NaCl, 2 mM EDTA, 0.1% NP40, 10% glycerol) and once in PBS. Precipitated proteins were then analyzed by immunoblotting or mass spectrometry.

#### NF-**κ**B reporter assay

Indicated cell lines were transfected using lipofectamine 3000 with 3 μg of p65 reporter plasmid (pHAGE NF-κB-TA-LUC-UBC-GFP-W plasmid from Addgene #49343). After 48 h, cells were lysed using 150 μl of Luciferase cell culture lysis buffer (Promega) and luminescence was assessed using 96-wells white plate. Luciferase activities were directly measured using Luciferase assay system (Promega) on TECAN infinite 200 PRO plate reader. GFP fluorescence, which is constitutively expressed by the reporter vector served to normalize the luminescent signal according to the transfection efficiency, and the p65 activity signals was depicted as luminescent intensity (reporter assay) / Fluorescent signal (GFP).

#### *In vitro* ubiquitin conjugation assays

HEK/293T cells were transfected in the presence of full length or truncated (1-175) CD95 constructs. After 24 h, cells were lysed in TNET buffer (50 mM Tris pH7.4, 100 mM NaCl, 5 mM EDTA, 0,5% Triton X-100, protease inhibitors) and CD95 was immunoprecipitated using the anti-CD95 mAb APO1-3. After washes in TNET buffer, beads were incubated in the presence of recombinant human p105 (150 ng) (Novus Biologicals) for 1 h at 37°C with 50 μl of ubiquitination reaction buffer [5 mM Mg-ATP solution, ubiquitin activating enzyme E1 (100 nM, Enzo Life Sciences, Villeurbanne, France), E2 UbcH5c (2.5 μM, Enzo Life Sciences), biotinylated Ubiquitin (2.5 μM, Enzo Life Sciences), 2U inorganic pyrophosphatase (Sigma Aldrich), 1 mM DTT]. The ubiquitination reaction was then stopped by the addition of 5X Laemmli buffer and samples were resolved by SDS-PAGE.

#### Protein-fragment complementation assay (PCA)

HEK/293T cells were electroporated with 10 μg of DNA using BTM-830 electroporation generator (BTX Instrument Division, Harvard Apparatus). 24 h after transfection, cells (10^6^) were washed and resuspended in 100 μl PBS placed in OptiPlate-96 (PerkinElmer, Waltham, MA, USA) in the presence or absence of indicated peptides (50 μM) for 1 hour. Coelenterazine-h (5 μM, Sigma-Aldrich) was then injected and the Renilla luciferase activity was assessed for the first 10 seconds using Infinite200Pro (Tecan, Männedorf, Switzerland).

#### Microarray analysis

RNA quality was assessed using an RNA6000 nano chip (Agilent). For each condition, 9 ng of RNA were reverse transcribed using the Ovation PicoSL WTA System V2 (Nugen, Leek, The Netherlands). MDA-MB-231 fragmented cDNAs were hybridized to GeneChip Human Gene 2.0 ST microarrays (Affymetrix). 4T1 fragmented cDNAs were hybridized to Clariom S mouse microarrays (Affymetrix). Hybridized microarrays were scanned by a GeneChip Scanner 30007G (Affymetrix). Raw data and quality-control metrics were generated using Expression Console software (Affymetrix). Raw CELs files were read with R in order to form two distinct “GeneFeatureSet” R object then normalized separately by robust multi-array averaging via rma function from oligo R package (Carvalho and Irizarry, 2010) with the “target=core” option to produce summaries at core gene level. Probes from the two resulting tables were mapped to corresponding gene symbol using hugene20sttranscriptcluster.db and clariomsmousetranscriptcluster.db from bioconductor as database for MDA-MB-231 and 4T1 datasets, respectively. Probes without attributed gene symbol were discarded and duplicate gene symbol mean was calculated.

For the parent and CD95 k.o. TNBC cells differential expression analysis, the two normalized datasets were merged by upper case gene symbol (human gene symbol format), where resulting line containing NA was dropped and a batch correction were made by ComBat function from Surrogate Variable Analysis R package (Leek et al., 2012). Raw and normalized data are deposited in the GEO database under accession ID GSE172215. For the three distinct analyses, an appropriate contrast matrix reflecting the genotype of the samples, CD95 k.o or WT was created to perform Statistical analyses using the Linear Model for Series of Arrays (lmFit) from limma R package (Ritchie et al., 2015) following by an expended fit step with “contrasts.fit” function on that matrix and the results was computed with “eBayes” function to make empirical Bayes statistics. For analysis of 4T1 and MDA-MB-231 separately, genes showing a minimum of 1.5 fold change in expression and a minimum P value of 0.05 were considered significant. For merged dataset analysis, genes showing a minimum of 1.5 fold change in expression and a minimum P value of 0.1 were considered significant.

#### GSEA

Gene Set Enrichment analysis was performed by GSEA function from ClusterProfiler R (Yu et al., 2012) package with Homo sapiens hallmark gene sets data previously collected in MSigDB R package, gene symbol translated into entrezid using bitr function with org.Hs.eg.db and pvalue cut off was considered significant at 0.25.

### Quantification and statistical analysis

All experiments were performed in triplicate. The results were expressed as mean ± SD and analyzed using unpaired and non-parametric Mann-Whitney t-test (Prism8). Statistical significance was defined as p < 0.05.

### Additional resources

This study has not generated or contributed to a new website/forum and is not part of a clinical trial.

## Data Availability

Sequencing data have been deposited in the GEO database :GSE172215. Proteomic data has been deposited to the ProteomeXchange via the PRIDE partner repository: PXD027196. This paper does not report original code. Any additional information required to reanalyze the data reported in this paper is available from the lead contact upon request.

## Supplemental information

Document S1. Figures S1 and S2

Table S1. Gene differently expressed between CD95 k.o. and wild-type MDA-MB-231 cells, related to Figure 1 Genes shown with p < 0.05, logFC cutoff 0.6.

Table S2. Gene differently expressed between CD95 k.o. and wild-type 4T1 cells, related to Figure 1 Genes shown with p < 0.05, logFC cutoff 0.8.

Table S3. Gene differently expressed between CD95 k.o. and wild-type TNBC cells (4T1 and MDA-MB-231 cells), related to Figure 1 Genes shown with p < 0.1, logFC cutoff 0.6.

Table S4. Cytokine expression in CD95 k.o. and wild-type TNBC cells, related to Figure 1 The expression level of the indicated cytokines was quantified using Proteome Profiler Human XL Cytokine array (see STAR Methods session). For each experiment, membrane dots have been quantified by densitometry to calculate mean and SD of three experiments performed independently.

Table S5. Proteomic analysis of BioID, related to Figure 2 Biotin-labeled proteins were pulled down in HEK/293T cells transfected with WT (CD95(WT)-BirA) or Mut2 corresponding to either a CD95-BirA construct devoid of its intracellular region (CD95(1–175)-BirA) or the CD95 transmembrane domain fused to BirA (TM-BirA)). Protein complexes were analyzed by MS/MS. The proteins enriched in CD95(WT)-BirA HEK/293T cells are depicted with the WT/mut2 and WT/BirA ratios.

Table S6. QPCR-primer sets, related to STAR Methods

Table S7. Cloning strategy – vectors and primers, related to STAR Methods
