# Supplementary material for: CD95/Fas suppresses NF-κB activation through recruitment of KPC2 in a CD95L/FasL-independent mechanism
Source: iScience. 2021 Dec 1;24(12):103538. doi: 10.1016/j.isci.2021.103538 (PMC8666665; doi:10.1016/j.isci.2021.103538)
Supplement: Document S1. Figures S1 and S2 [file mmc1.pdf]

iScience, Volume *24*

Supplemental information

CD95/Fas suppresses NF-κB activation through recruitment of KPC2 in a CD95L/FasL-independent mechanism

Jean-Philippe Guégan, Justine Pollet, Christophe Ginestier, Emmanuelle Charafe-Jauffret, Marcus E. Peter, and Patrick Legembre

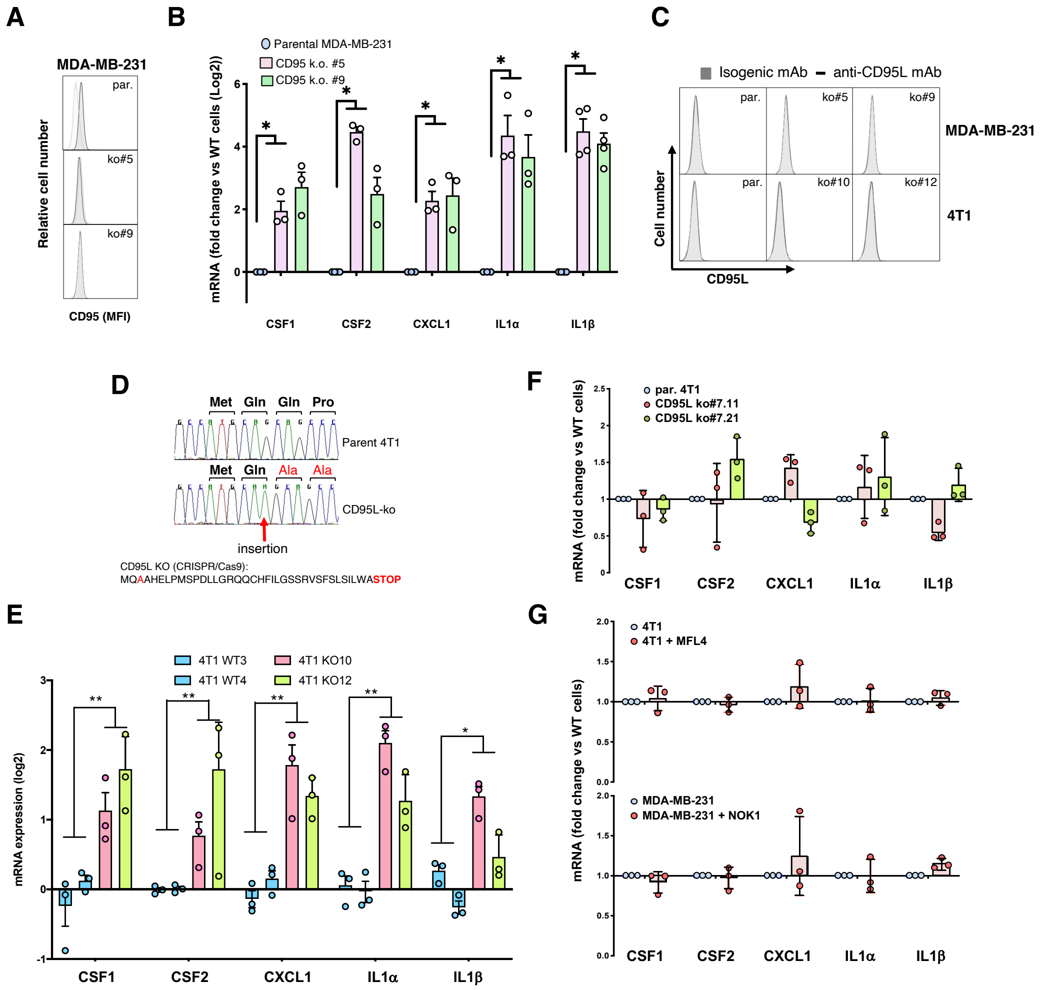

Figure S1, Related to Figure 1. Generation and characterization of CD95 k.o. TNBC cells.

(A) Using flow cytometry, CD95 expression was assessed in parental TNBC cells (MDA-MB-231) and their counterparts in which CD95 was knocked-out using CRISPR/Cas9 method. Light grey histograms represent the isotypic control staining.

(B) The expression level of the five-cytokines signature was compared between the parental MDA-MB-231 cell line and two CD95 k.o. counterparts using qPCR. Data are represented as normalized gene expression (fold change; Log(2)) compared to the parental cell line and represent mean and SD of at least three independently performed experiments (n=3 and n=4 for IL1β).

(C) CD95L expression was assessed by flow cytometry in indicated cells.

(D) CRISPR/Cas9 was used to generate CD95L k.o. in 4T1 cells. Cells were cloned and CD95L DNA was sequenced. An alanine was inserted in the CD95L k.o. 4T1 TNBC cells creating a frameshift mutation that leads to a shortened polypeptide.

(E) Five-cytokines signature expression was compared in two wild-type (WT) and two CD95 k.o. 4T1 cells using qPCR. Data represent mRNA expression level (Log(2)) and represent mean and SD of three independently performed experiments.

(F) Five-cytokines signature expression was quantified in parental (par.) and CD95L k.o. 4T1 cells using qPCR. Data are represented as normalized gene expression (fold change) compared to parental cells and represent mean and SD of three independently performed experiments.

(G) Five-cytokines signature expression was quantified in 4T1 (mouse) or MDA-MB-231 (human) WT cells incubated in the presence or absence of the neutralizing anti-mouse CD95L mAb MFL4 (*upper panel*) and the neutralizing anti-human CD95L mAb NOK1 (*lower panel*), respectively using qPCR. Data are represented as normalized gene expression (fold change) compared to wild-type cells and represent mean and SD of three independently performed experiments.

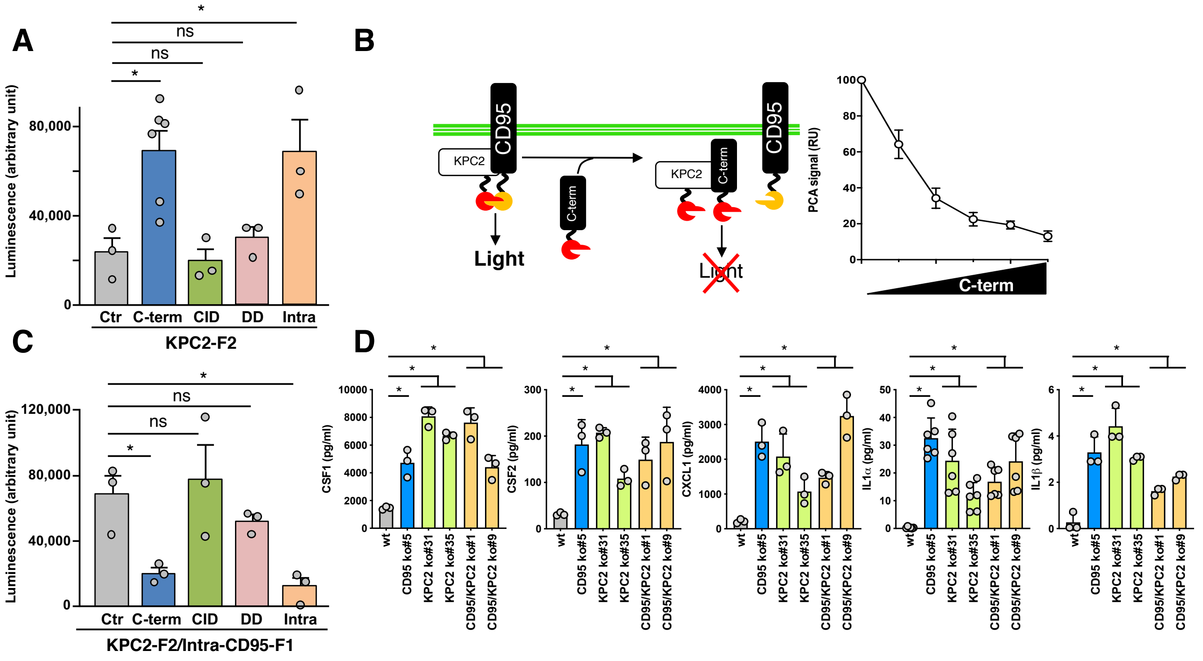

Figure S2, Related to Figures 3 and 4. KPC2 directly interacts with the C-terminal domain of CD95.

(A) HEK/293T cells were co-transfected with plasmids encoding KPC2 and CD95 domains fused to the F2 domain and with the F1 fragment of Renilla Luciferase, respectively and after 24 hours, luminescence was assessed. Data represent mean and SD (n=3-6). * stands for $p<0.05$ using unpaired and non-parametric Mann-Whitney t-test.

(B) *Left panel:* Schematic representation of the PCA competitive assay. *Right panel:* HEK/293T cells were co-transfected with three plasmids encoding KPC2 and CD95 domains fused to the F2 domain and with the F1 fragment of Renilla Luciferase, respectively and C-term CD95-F2 transfected at different quantities and after 24 hours, luminescence was assessed.

(C) HEK/293T cells were transfected with plasmids encoding KPC2-F2 and the whole intracellular region of CD95 (175-319)-F1. A PCA competitive assay was developed by co-transfecting indicated F2-fused proteins and the inhibition of luminescence was assessed. Data represent mean and SD of three independently performed experiments. * stands for $p<0.05$ using unpaired and non-parametric Mann-Whitney t-test.

(D) Inflammatory cytokines produced for 4 days by indicated MDA-MB-231 cells were quantified by ELISAs. Data represent mean and SD (n=3-6). * stands for $p<0.05$ using unpaired and non-parametric Mann-Whitney t-test.
